# Binning a continuous trait: Effects on genetic evaluation and selection

**DOI:** 10.3168/jdsc.2026-1096

**Published:** 2026-10-05

**Authors:** Xiao-Lin Wu, Guilherme J.M. Rosa, Allicia Horn, Ezequiel Nicolazzi, John B. Cole

**Affiliations:** 1 Council on Dairy Cattle Breeding, Bowie, MD 20716; 2 Department of Animal and Dairy Sciences, University of Wisconsin, Madison, WI 53706; 3 Brown Swiss Association, Beloit, WI 53511; 4 Department of Animal Sciences, Donald Henry Barron Reproductive and Perinatal Biology Research Program, and the Genetics Institute, University of Florida, Gainesville, FL 32611; 5 Department of Animal Science, North Carolina State University, Raleigh, NC 27695; 6 Department of Animal Biosciences, University of Guelph, Ontario, Canada N1G 2W1

## Abstract

Binning continuous phenotypes into discrete categories is a longstanding practice in dairy cattle evaluation and management, yet its effects on genetic evaluation and selection remain insufficiently characterized. We develop a theoretical framework to investigate how binning affects heritability, EBV, and selection response. By expressing the discretized phenotype as a linear projection of the underlying continuous phenotype, we show that binning is governed by 2 quantities: an attenuation factor that scales the genetic signal and a variance rescaling term. These 2 components jointly determine the reduction in heritability, the degree of shrinkage in mixed-model equations, and EBV reliability. Coarse discretization, such as binary traits, results in substantial losses of genetic information and selection response. In contrast, moderate binning (e.g., 7–10 categories) retains most of the underlying signal, with only minor reductions in accuracy. Further increases in the number of categories yield diminishing returns, as genetic parameters and selection efficiency converge rapidly to those of the continuous trait. Selection response is relatively more robust to discretization than variance-based measures, indicating that individual rankings are largely preserved with a sufficient number of categories. These findings provide practical guidance for the design and interpretation of discretized traits in dairy genetic evaluations, highlighting the trade-off between recording simplicity and information retention.

## Graphical Abstract

**Figure:**
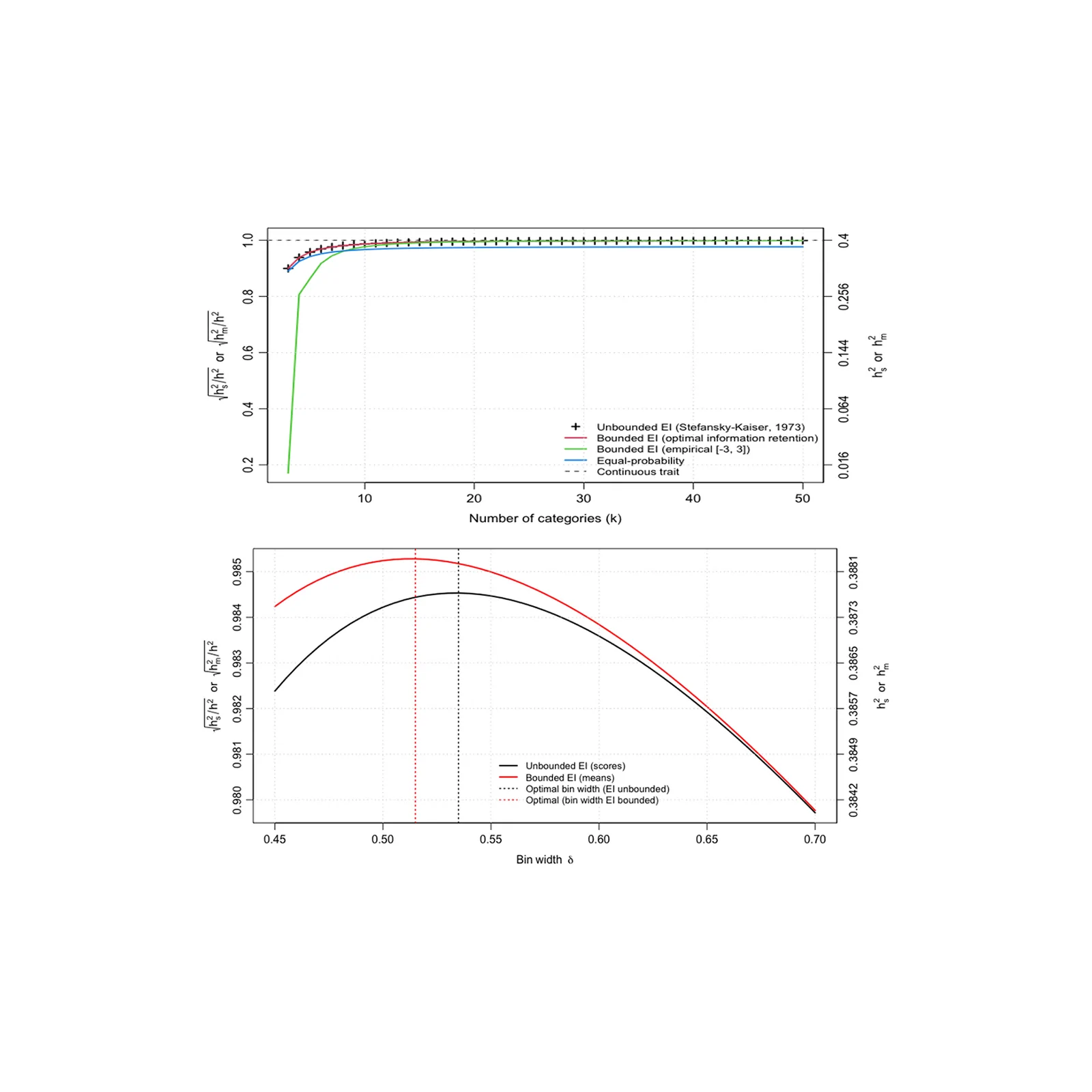

Continuous biological traits are often discretized into ordinal scores (e.g., 1–9 or 1–50 linear-type scales) in dairy genetic evaluations and management (Veerkamp and Brotherstone, 1997; Manafiazar et al., 2016; Wu et al., 2026a). However, binning a continuous variable inevitably leads to information loss (Cox, 1957). This occurs because discrete categories reduce resolution, limiting the ability to capture subtle but potentially important variation in the data. Additionally, the variance of the resulting discrete scores can differ substantially from that of the original measurements because the 2 scales are not directly comparable, raising concerns for analyses that require compatible variance-covariance structures (Wu et al., 2023).

In a previous study (Wu et al., 2026b), we evaluated the principles and methods for binning a continuous trait from a phenotypic analysis perspective, focusing on the optimal number of bins and bin width under equal-interval binning. We showed that the optimal strategy depends critically on the criterion used to quantify agreement or information retention, which is tied to specific analytical objectives.

This study extends that work by examining how binning affects genetic evaluations and selection. Two binning methods are considered: equal interval (**EI**) and equal probability (**EP**). We examine the effect of the number of categories under both methods and assess bin width choice within the EI framework. We also compare ordinal scores and bin-specific conditional means as phenotypes in genetic evaluations under both methods. Liability (threshold) models are not considered because they are typically applied to binary traits or traits with relatively few categories, and are rarely used for linear-type (conformation) traits with 9 or more categories.

Let a continuous phenotype (*y_i_*) be modeled as follows:

[1] $$y_{i}=\mu +{z^{\prime }}_{i}a+e_{i},$$

where *µ* is the overall mean, $a∼N\left(0,A\sigma_{u}^{2}\right)$ is a vector of additive genetic effects with mean vector zero and variance-covariance matrix $A\sigma_{u}^{2},$ $\sigma_{u}^{2}$ is the additive genetic variance, **A** is numerator additive genetic relationship matrix, **z***_i_* is the incidence vector linking observation *i* to the additive genetic effects, and $e_{i}∼N\left(0,\sigma_{e}^{2}\right)$ is the corresponding residual with zero mean and variance $\sigma_{e}^{2}.$ For simplicity, we assume that the phenotype has been adjusted for all fixed effects except the overall mean and standardized so that Var(*y_i_*) = 1, $\sigma_{a}^{2}=h^{2},$ and $\sigma_{e}^{2}=1-h^{2}.$ Results on the original scale can be recovered by applying the inverse transformation.

Let the phenotypes be divided into ordered categories using the following thresholds:

$$\text{\textbackslash{}[}-\infty =\tau_{0}<\tau_{1}<\cdots <\tau_{k-1}<\tau_{k}=\infty .\text{\textbackslash{}]}$$

The traditional, unbounded EI binning (Cox, 1957) defines the interior bin thresholds as

[2] $$\text{\textbackslash{}[}\tau_{j}=\left(j\frac{k}{2}\right)\delta ,j=1,\ldots ,k-1,\text{\textbackslash{}]}$$

where *δ* > 0 is the common bin width.

In contrast, the interior thresholds for bounded EI binning are defined (Wu et al., 2026b) as

[3] *τ_j_* = −Λ + (*j* − 1)*δ*, for *j* = 1, . . . , *k* − 1,

where $\delta =\frac{2\wedge }{k-2},$ or equivalently $\wedge =\frac{k-2}{2}\delta .$ The 2 extreme bins correspond to (−∞,−∧) and [∧,∞]. The parameter Λ can be determined either empirically, for example, Λ = 3, which covers ∼99.7% of a standard normal distribution, or optimally by maximizing information retention (Wu et al., 2026b).

Given the interior thresholds, the categorical scores are defined as

*s_i_* = *j* if *τ_j_*_−1_ < *y_i_* ≤ *τ_j_*, *j* = 1, . . . , *k*.

The category probabilities are

[4] *p_j_* = Pr(*s_i_* = *j*) = Φ(*τ_j_*) − Φ(*τ_j−1_*), *j* = 1, . . . , *k*,

where Φ is the standard normal cumulative distribution function.

For EP binning, because *p*_1_ = . . . = *p_k_* = 1/*k*, the thresholds are defined as

[5] $$\tau_{j}=\Phi^{-1}\left(\frac{j}{k}\right)forj=1,\ldots ,k-1.$$

For all binning methods, the mean and variance of the discretized scores are

[6] $$E\left(s_{i}\right)=\frac{k+1}{2}\left(undersymmetry\right),$$

and

[7] $$Var\left(s_{i}\right)=\overset{k}{\underset{j=1}{\sum }}j^{2}p_{j}-{\left(\overset{k}{\underset{j=1}{\sum }}jp_{j}\right)}^{2}.$$

Specifically for EP, the equal-probability-bins property simplifies the variance to

[8] $$Var\left(s_{i}\right)=\frac{k^{2}-1}{12}.$$

For genetic evaluation, we fit the following linear mixed model to discrete scores, treating them as a continuous response variable:

[9] $$\text{\textbackslash{}[}s_{i}=\mu_{s}+z_{i}^{\prime }a_{s}+e_{s,i},\text{\textbackslash{}]}$$

where $a_{s}∼N\left(0,A\sigma_{a_{s}}^{2}\right)$ is a vector of additive genetic effects on the discrete score scale, and $e_{s,i}∼N\left(0,\sigma_{e_{s}}^{2}\right)$ is the residual term.

Wu et al. (2026a) proposed a linear projection approach to relate discretized scores to the original continuous measurements:

[10] *s_i_* = *γ_s_y_i_* + *η_s_*_,_*_i_*.

Here, the linear projection coefficient *γ_s_* quantifies the extent to which the latent continuous signal corresponds to the discretized score, and *η_s_*_,_*_i_* is the projection residual, which is assumed to be uncorrelated (orthogonal) to *y_i_*; that is, Cov(*η_s_*_,_*_i_*, *y_i_*) = 0. Any intercept term is absorbed into *η_s_*_,_*_i_*.

Because Var(*y_i_*) = 1 and E(*y_i_*) = 0,

[11] $$\text{\textbackslash{}[}\begin{array}{c} \gamma_{s}=\frac{Cov\left(s_{i},y_{i}\right)}{Var\left(y_{i}\right)}=Cov\left(s_{i},y_{i}\right)=E\left\{\left[s_{i}-E\left(s_{i}\right)\right]y_{i}\right\}=E\left(s_{i}y_{i}\right)=\\ =\sum_{j=1}^{k-1}\phi \left(\tau_{j}\right), \end{array}\text{\textbackslash{}]}$$

where *ɸ* is the standard normal probability density function. This formula holds for any threshold-based discretization under the standard normal distribution. The value of *γ_s_* differs between EI and EP binning.

Substituting Equation [1] into Equation [10] gives

[12] $$s_{i}=\gamma_{s}\mu +\gamma_{s}z_{i}^{\prime }a+\left(\gamma_{s}e_{i}+\eta_{s,i}\right).$$

Hence, after discretization, the genetic signal is attenuated (scaled) by *γ_s_*, and the residual term becomes a composite of the original residual and the discretization error.

Then, the additive genetic variance on the score scale, ignoring the additive genetic relationship matrix **A** for variance derivation, is

[13] $$\sigma_{a_{s}}^{2}=Var\left(\gamma_{s}a\right)=\gamma_{s}^{2}\sigma_{a}^{2}=\gamma_{s}^{2}h^{2}.$$

Because *η_s_*_,_*_i_* is orthogonal to *y_i_* and Var(*y_i_*) = 1, the total phenotypic variance becomes

$$Var\left(s_{i}\right)=\gamma_{s}^{2}Var\left(y_{i}\right)+Var\left(\eta_{s,i}\right)=\gamma_{s}^{2}+Var\left(\eta_{s,i}\right).$$

Thus, under pseudo-linear analysis of discretized scores, the implied score-scale heritability is

[14] $$h_{s}^{2}=\frac{\sigma_{a_{s}}^{2}}{Var\left(s_{i}\right)}=\frac{\gamma_{s}^{2}h^{2}}{\gamma_{s}^{2}+Var\left(\eta_{s,i}\right)}.$$

Thus, the reduction in $h_{s}^{2}$ relative to the underlying heritability h^2^ is governed by 2 components: (1) signal attenuation, captured by $\gamma_{s}^{2},$ and (2) variance rescaling, captured by $\text{\textbackslash{}[}Var\left(s_{i}\right)=\gamma_{s}^{2}+Var\left(\eta_{s,i}\right).\text{\textbackslash{}]}$

On the population level, denote

[15] **s** = **1***μ_s_* + **Za***_s_* + **e***_s_*,

where **s** is a vector of ordinal scores, *μ_s_* = *γ_s_μ* is the population mean, **a***_s_* = *γ_s_***a** is the vector of additive genetic effects on the ordinal-score scale with the corresponding incidence matrix **Z**, and **e***_s_* = *γ_s_***e** + **η***_s_* is a vector of residual effects, assumed to follow $N\left(0,I\sigma_{e_{s}}^{2}\right).$

The BLUP of breeding values on the score scale is

[16] $${\hat{a}}_{s}=AZ\prime {\left(ZAZ\prime +I\lambda_{s}\right)}^{-1}\left(s-1{\hat{\mu }}_{s}\right),$$

where

[17] $$\lambda_{s}=\frac{\sigma_{e_{s}}^{2}}{\sigma_{a_{s}}^{2}}=\frac{Var\left(s_{i}\right)-\gamma_{s}^{2}h^{2}}{\gamma_{s}^{2}h^{2}}.$$

For the original continuous trait,

[18] $$\lambda =\frac{\sigma_{e}^{2}}{\sigma_{a}^{2}}=\frac{1-h^{2}}{h^{2}}.$$

Therefore, binning affects EBV within the pseudo-linear framework by replacing

$$\lambda =\frac{1-h^{2}}{h^{2}}\to \lambda_{s}=\frac{Var\left(s_{i}\right)-\gamma_{s}^{2}h^{2}}{\gamma_{s}^{2}h^{2}}=\frac{1-\frac{\gamma_{s}^{2}}{Var\left(s_{i}\right)}h^{2}}{\frac{\gamma_{s}^{2}}{Var\left(s_{i}\right)}h^{2}}.$$

Because $\frac{\gamma_{s}^{2}}{Var\left(s_{i}\right)}$ is typically less than 1, the score-based analysis tends to have a larger variance ratio; that is, *λ_s_* > *λ*, implying greater shrinkage and less information for predicting breeding values.

In general, a precise closed-form identity such as **â***_s_* = *γ_s_***â** does not hold, because BLUP also depends on the variance ratio in the mixed-model equations. However, the score-based and continuous-trait predictors are linked through the following 2 BLUP equations:

**â** = **AZʹ**(**ZAZʹ** + **I***λ*)^−1^ (**y** − **1***μ*)

and

**â***_s_* = **AZʹ**(**ZAZʹ** + **I***λ_s_*)^−1^ (**s** − **1***μ_s_*).

Substituting **s** with **s** = *γ_s_***y** + **η***_s_* in the equation for **â***_s_* gives

[19] **â***_s_* ≈ **AZʹ**(**ZAZʹ** + **I***λ_s_*)^−1^ (*γ_s_***y** + **η***_s_* − **1***μ_s_*).

Thus, relative to **â**, the score-based EBV differs for 2 reasons: (1) signal attenuation $y\mapsto \gamma_{s}y,$ and (2) variance-ratio change $\lambda \mapsto \lambda_{s}.$ Therefore, the effect of binning is not a simple rescaling by *γ*, but rather a combination of signal attenuation and increased shrinkage.

The accuracy of EBV on the score scale is expressed as

[20] $$\begin{array}{c} Corr\left({\hat{a}}_{s,i},a_{s,i}\right)=\frac{Cov\left({\hat{a}}_{s,i},a_{s,i}\right)}{\sqrt{Var\left({\hat{a}}_{s,i}\right)Var\left(a_{s,i}\right)}}=\\ \frac{Var\left({\hat{a}}_{s,i}\right)}{\sqrt{Var\left({\hat{a}}_{s,i}\right)Var\left(a_{s,i}\right)}}=\sqrt{\frac{Var\left({\hat{a}}_{s,i}\right)}{Var\left(a_{s,i}\right)}}, \end{array}$$

because *â_s_*_,_*_i_* is the linear projection of *a_s_*_,_*_i_.*

Because the prediction error *â_s_*_,_*_i_* − *a_s_*_,_*_i_* is assumed to be uncorrelated with *â_s_*_,_*_i_*,

Var(*a_s_*_,_*_i_*) = Var(*â_s_*_,_*_i_*) + Var(*â_s_*_,_*_i_* − *a_s_*_,_*_i_*).

Thus,

Var(*â_s_*_,_*_i_*) = Var(*a_s_*_,_*_i_*) − PEV*_s_*_,_*_i_*,

where PEV*_s_*_,_*_i_* is the prediction error variance: PEV*_s_*_,_*_i_* = Var(*â_s_*_,_*_i_* − *a_s_*_,_*_i_*). Therefore, assuming that *A_ii_* = 1 and that reliability is defined with respect to the same target breeding value,

[21] $$Corr\left({\hat{a}}_{s,i},a_{s,i}\right)=\sqrt{\frac{Var\left(a_{s,i}\right)-PEV_{s,i}}{Var\left(a_{s,i}\right)}}=\sqrt{Rel_{s,i}}.$$

Because correlation is invariant to linear scaling, and **a***_s_* ≈ *γ_s_***a** under moderate-to-fine binning, it follows that

[22] $$Corr\left({\hat{a}}_{s,i},a_{i}\right)\approx Corr\left({\hat{a}}_{s,i},a_{s,i}\right)=\sqrt{Rel_{s,i}}.$$

The effect of binning on selection can be described directly using the breeder's equation (Falconer and Mackay, 1996). When selection is based on EBV, the expected population response on the underlying continuous-trait scale is

[23] *R_s_* = *ir_s_σ_a_*,

where *i* is the selection intensity, *r_s_* = Corr(*â_s,_ a*) is the selection accuracy, and $\sigma_{a}=\sqrt{Var\left(a\right)}$ is the additive genetic SD on the original continuous scale.

By applying Equations [21] and [22],

$$R_{s}\approx i\;\sigma_{a}\;\sqrt{{\overset{―}{Rel}}_{s}},$$

where ${\overset{―}{Rel}}_{s}$ denotes the average reliability across the selected individuals.

For the original continuous phenotype,

$$R=i\;\sigma_{a}\;\sqrt{\overset{―}{Rel}}.$$

Comparing the 2 expressions shows that the proportional loss in expected selection response from binning is

[24] $$\frac{R_{s}}{R}\approx \sqrt{\frac{{\overset{―}{Rel}}_{s}}{\overset{―}{Rel}}}.$$

For the repeated-record approximation (Falconer and Mackay, 1996),

$$Rel=\frac{nh^{2}}{1+\left(n-1\right)h^{2}}andRel_{s}=\frac{nh_{s}^{2}}{1+\left(n-1\right)h_{s}^{2}},$$

then

[25] $$\frac{R_{s}}{R_{}}\approx \sqrt{\frac{nh_{s}^{2}}{1+\left(n-1\right)h_{s}^{2}}\times \frac{1+\left(n-1\right)h^{2}}{nh^{2}}}.$$

When *n* = 1, this reduces to

[26] $$\frac{R_{s}}{R_{}}\approx \sqrt{\frac{h_{s}^{2}}{h^{2}}}=\sqrt{\frac{\gamma_{s}^{2}}{Var\left(s_{i}\right)}}.$$

Alternatively, each observation can be assigned the conditional mean of the underlying continuous trait within its category. Let *B_j_* denote the interval defined by thresholds *τ_j_*_−1_ < *y_i_* ≤ *τ_j_*, and define the grouped phenotype as

[27] *m_i_* = E(*y_i_* | *B_j_*).

Hence, *m_i_* is the conditional expectation of *y_i_* given its category, and therefore represents the optimal least-squares approximation to *y_i_* based on the discretized information.

The linear projection of *m_i_* onto *y_i_* can be written as

[28] *m_i_* = *γ_m_y_i_* + *η_i_*,

where $\gamma_{m}=\frac{Cov\left(m_{i},y_{i}\right)}{Var\left(y_{i}\right)}$ = Cov(*m_i_*, *y_i_*). Because *m_i_* = E(*y_i_* | *B_j_*), it follows that Cov(*m_i_*, *y_i_*) = Var(*m_i_*), and therefore

[29] *γ_m_* = Var(*m_i_*).

Noting that *γ_m_* = Var(*m_i_*) ≈ $\frac{\gamma_{s}^{2}}{Var\left(s_{i}\right)},$ the information retention for ordinal phenotypes can be quantified as

[30] $$M=\frac{\gamma_{s}^{2}}{Var\left(s_{i}\right)}.$$

The additive genetic variance on the conditional mean scale becomes

[31] $$\sigma_{a_{m}}^{2}=\gamma_{m}^{2}\sigma_{a}^{2}.$$

The corresponding heritability is

[32] $$h_{m}^{2}=\frac{\sigma_{a_{m}}^{2}}{Var\left(m_{i}\right)}=\gamma_{m}h^{2}.$$

Thus, with conditional means as phenotypes, heritability is reduced in proportion of phenotypic variance retained after grouping. Unlike ordinal scores, no additional variance inflation occurs, and the grouped phenotype provides the most efficient representation of the continuous trait within each category.

With *m_i_* as the continuous response variable, the linear mixed model is

[33] **m** = **1***μ_m_* + **Za***_m_* = *e_m_*.

The BLUP of breeding value on the conditional mean scale is

[34] **â***_m_* = **AZʹ**(**ZAZʹ** + **I***λ_m_*)^−1^ (**m** − **1***μ_m_*),

where the variance ratio is

[35] $$\lambda_{m}=\frac{\sigma_{e_{m}}^{2}}{\sigma_{a_{m}}^{2}}=\frac{1-h_{m}^{2}}{h_{m}^{2}}=\frac{1-\gamma_{m}h^{2}}{\gamma_{m}h^{2}}.$$

Selection efficiency with conditional means as the response is assessed for ordinal scores, with appropriate substitutions replacing $\left(s_{i},h_{s}^{2},\lambda_{s}\right)$ with $\left(m_{i},h_{m}^{2},\lambda_{m}\right).$

Table 1 summarizes the effects of the finite number of categories (*K* = 2, 9, and 50) on heritability, variance ratio, and relative selection efficiency (**RSE**) under different binning strategies, where *y_i_* ∼ *N*(0,1) and h^2^ = 0.4. Relative selection efficiency is defined as hs2/hs2h2h2 for ordinal scores and as hm2/hm2h2h2 for conditional means. Optimal bin widths are derived under bounded EI (Wu et al., 2026b) and under unbounded EI (Stefansky and Kaiser, 1973). Bin widths under EP are implicitly determined by the quantiles of the standard normal distribution.

**Table 1:** Effects of binning on variance ratio, heritability estimate, and relative selection efficiency (RSE)^1^, ^2^, ^3^, ^4^

*k*	Method	Scale	Varp	*γ_s_* (*γ_m_*)	$h_{s}^{2}$$\left(h_{m}^{2}\right)$	*λ_s_* (*λ_m_*)	RSE
2	EI/EP	1–2	0.250	0.399	0.255	2.930	0.798
	EI (unbounded)	1–9	3.400	1.815	0.388	1.580	0.985
9	EI (unbounded)	5 × 1–9	84.99	9.076	0.388	1.580	0.985
	EI (bounded)	1–9	3.629	1.875	0.388	1.580	0.985
	EI (bounded)	E(*y_i_* | *B_j_* = 1:9)	0.971	0.971	0.388	1.575	0.985
	EI (empirical)	1–9	1.443	1.166	0.377	1.653	0.971
	EP	1–9	6.667	2.492	0.373	1.680	0.967
50	EI (unbounded)	1–50	60.00	7.740	0.399	1.500	0.999
	EI (bounded)	1–50	65.87	8.109	0.399	1.500	0.999
	EI (bounded)	E(*y_i_* | *B_j_* = 1:50)	0.999	0.999	0.399	1.500	0.999
	EI (empirical)	1–50	63.83	7.983	0.399	1.504	0.999
	EP	1–50	208.3	14.09	0.382	1.620	0.977

1 *k* = number of categories; Varp = variance of phenotypes, defined as group means [E(*y_i_* | *B_j_* = 1:9)] or ordinal scores; *γ_s_* (*γ_m_*) = linear projection coefficient of ordinal-score (group-mean) phenotypes on the latent continuous phenotype; $h_{s}^{2}$$\left(h_{m}^{2}\right)$ = heritability on the ordinal-score (group-mean) scale; *λ_s_* (*λ_m_*) = ratio of residual variance to genetic variance on the ordinal-score (group-mean) scale.

2 5 × 1–9 denotes a linearly rescaled version obtained by multiplying each score by 5 (yielding scores of 5, 10, ..., 45).

3 For the continuous trait, h^2^ = 0.4 and $\lambda =\frac{1-0.4}{0.4}$ = 1.50.

4 EI = equal interval; EP = equal probability.

Binary scores (*k* = 2) cause substantial information loss. The heritability decreases from 0.40 to 0.255, a reduction of ∼36%, and the variance ratio increases markedly from 1.50 to 2.93, indicating considerably greater shrinkage in the mixed-model equations. As a result, RSE declines by ∼20% relative to the continuous-scale response. Very coarse discretization can severely compromise both parameter estimation and selection performance.

In contrast, moderate discretization with *k* = 9 categories preserves most of the genetic signal. For both unbounded and bounded EI approaches, heritability is 0.388, close to 0.40, and the variance ratio increases only slightly from 1.50 to ∼1.58. The corresponding RSE is ∼0.985, indicating that selection efficiency is mostly retained. Notably, these results remain essentially unchanged when ordinal scores (1–9) are multiplied by 5 (yielding scores of 5, 10, . . ., 45), reflecting the invariance of heritability and RSE to linear rescaling.

This rescaling was intended to approximate the variance of finer scoring systems (e.g., 1–50 scales). However, the resulting variance (≈85.0) substantially exceeds that of 1–50 (≈60.0). Given Var(*s_i_*) = 3.40 for *k* = 9, matching a target variance of 60.0 would require a scaling factor of approximately $\sqrt{60.0/3.40}$ ≈ 4.2 or slightly less, rather than 5.

Using conditional means as phenotypes under bounded EI binning yields identical heritability and RSE as ordinal scores and a slightly smaller variance ratio (1.575 vs. 1.580), indicating less shrinkage and slightly more precise EBV. Although the numerical difference is small in this example, the grouped-mean approach has the conceptual advantage of providing the least-squares-optimal representation of the underlying continuous trait within each category.

In contrast, the empirical bounded EI approach with a fixed span (−3, +3) performs noticeably worse at *k* = 9. The heritability decreases to 0.377, the variance ratio increases to 1.653, and the RSE declines to 0.971, indicating that the empirically chosen bin boundaries are suboptimal for moderate numbers of categories.

Equal-probability binning yields lower efficiency at *k* = 9 than EI. Although *γ_s_* is larger under EP, the variance of the ordinal scores is substantially inflated (6.667), leading to lower heritability (0.373) and a higher variance ratio (1.680). Consequently, the RSE decreases to 0.967.

For fine discretization (*k* = 50), all EI binning approaches produce results virtually identical to those of the continuous trait. The heritability approaches 0.399, the variance ratio converges to ∼1.50, and the RSE reaches 0.999, indicating negligible information loss. Sufficiently fine discretization effectively recovers the original continuous-scale properties. The EP binning also improves at *k* = 50, but retains a small deficit, with a lower heritability of 0.382 and a smaller RSE of 0.977.

The heritability estimate $\left(h_{s}^{2}{orh}_{m}^{2}\right)$ increases rapidly as the number of categories (*k*) rises from 3 to about 10, then gradually plateaus toward the underlying continuous-scale heritability (h^2^ = 0.40; Graphical Abstract, upper panel). Both bounded and unbounded EI binning curves are nearly indistinguishable across all *k*, indicating that boundary treatment has minimal effect on information retention with many categories. Even with a moderate number of categories (e.g., *k* = 7–9), $h_{s}^{2}\left(h_{m}^{2}\right)$ already approaches 95% to 97% of the target value. Beyond *k* ≈ 15, further increases in *k* yield only marginal gains, reflecting diminishing returns from finer discretization.

Equal-probability binning produces lower heritability across all *k*, because it inflates the variance of discrete scores, particularly with a large *k*, reducing the key ratio γs2/γs2Var(si)Var(si) that governs $h_{s}^{2}.$ Although the EP curve also converges toward the continuous-trait heritability as *k* increases, it does so more slowly than EI on the ordinal scale. Nonetheless, using group means as the response under finer EP binning yields results highly comparable to EI.

The RSE, measured as hs2/hs2h2h2 or hm2/hm2h2h2, exhibits a similar but more rapid recovery pattern as the number of categories increases. Relative efficiency increases sharply from *k* = 3 and quickly approaches unity, indicating that the ability to rank individuals correctly is largely preserved even under moderate discretization. The curve becomes nearly flat for *k* > 15, suggesting that additional categories provide negligible improvements in selection performance.

For sufficiently fine discretization (e.g., *k* ≥ 30), EI methods effectively recover the continuous-trait analysis, with $h_{s}^{2}\to h^{2}$ $\text{\textbackslash{}[}\left(h_{m}^{2}\to h^{2}\right)\text{\textbackslash{}]}$ and hs2/hs2h2h2→1 (hm2/hm2h2h2→1),  indicating negligible information loss, whereas EP converges more slowly on the ordinal scale, retaining a small but noticeable deficit even at larger *k*. Approximately 7 to 9 categories are sufficient to retain at least 95% of the selection efficiency under EI, whereas EP requires more categories (≈9–10) to reach the same level. To achieve 99% target selection efficiency, ∼18 to 20 categories are required under EI and 25 to 30 under EP.

Finally, the influence of bin width choice on genetic evaluation and selection is shown for *k* = 9 under unbounded EI (Graphical Abstract; lower panel). As *δ* increases from 0.45, both the heritability estimate $\text{\textbackslash{}[}(h_{s}^{2}\;\text{\textbackslash{}]}$ or $\text{\textbackslash{}[}\;h_{m}^{2})\text{\textbackslash{}]}$ and RSE (hs2/hs2h2h2 or hm2/hm2h2h2) increase initially, reflecting improved coverage of the underlying distribution and better alignment between the discretized and continuous phenotypes. They peak near the optimal bin width, approximately *δ* = 0.513 under bounded EI and *δ* = 0.534 under unbounded EI. Afterward, further increases in *δ* lead to a gradual decline as the bins become overly wide and resolution is lost. This pattern highlights the fundamental trade-off between insufficient span coverage at small *δ* and excessive coarsening at large *δ*. The optimal bin width for *k* = 9 derived from the genetic evaluation framework agrees with those obtained from phenotypic analysis (Wu et al., 2026b), and they are intrinsic to binning strategies.

Because RSE is a monotonic transformation of heritability, both measures peak at the same number of categories or bin width, confirming that the binning configuration that maximizes phenotypic information retention also maximizes downstream genetic efficiency. The relatively flat peak around the optimum indicates that moderate deviations from the optimum have only a small influence on heritability and selection response, suggesting robustness in practical applications. Compared with the empirical bin width, carefully calibrated bin widths are important for optimizing both statistical and genetic performance.

Overall, the number of categories is the primary determinant of information retention, with most gains achieved by increasing *k* from very small values to moderate levels (∼7–10 categories). Equal-interval binning consistently outperforms EP binning for moderate *k*, particularly under empirical approaches, its influence diminishes as the number of categories increases. Although discretization reduces variance-based measures such as heritability, its effect on selection efficiency is relatively smaller, indicating that the ranking of individuals is largely preserved under moderate binning. These findings highlight that the consequences of binning depend critically on its resolution and implementation, and they provide practical guidance for balancing the simplicity of recording with the preservation of genetic information in dairy cattle evaluations.
